# The “Coagulation Box” and a New Hemoglobin-Driven Algorithm for Bleeding Control in Patients with Severe Multiple Traumas

**DOI:** 10.5812/atr.10894

**Published:** 2013-06-01

**Authors:** Peter Hilbert, Gunther Olaf Hofmann, Jörg Teichmann, Manuel F. Struck, Ralph Stuttmann

**Affiliations:** 1 Department of Anesthesiology, Intensive Care and Emergency Medicine, BG-Kliniken Bergmannstrost, Halle, Germany; 2 Department of Trauma and Reconstructive Surgery, Friedrich-Schiller-University Jena, BG-Kliniken Bergmannstrost, Halle, Germany; 3 Department of Pharmacy, BG-Kliniken Bergmannstrost, Halle, Germany; 4 Department of Anesthesiology and Intensive Care Medicine, University Hospital Leipzig, Leipzig, Germany

**Keywords:** Trauma, Hemorrhage, Coagulation Disorder, Bleeding Control, Blood Coagulation Factors

## Abstract

**Background:**

Extensive hemorrhage is the leading cause of death in the first few hours following multiple traumas. Therefore, early and aggressive treatment of clotting disorders could reduce mortality. Unfortunately, the availability of results from commonly performed blood coagulation studies are often delayed whereas hemoglobin (Hb) levels are quickly available.

**Objectives:**

In this study, we evaluated the use of initial hemoglobin (Hb) levels as a guide line for the initial treatment of clotting disorders in multiple trauma patients.

**Patients and Methods:**

We have developed an Hb-driven algorithm to initiate the initial clotting therapy. The algorithm contains three different steps for aggressive clotting therapy depending on the first Hb value measured in the shock trauma room, (SR) and utilizes fibrinogen, prothrombin complex concentrate (PCC), factor VIIa, tranexamic acid and desmopressin. The above-mentioned drugs were stored in a special “coagulation box” in the hospital pharmacy, and this box could be immediately brought to the SR or operating room (OR) upon request. Despite the use of clotting factors, transfusions using red blood cells (RBC) and fresh frozen plasma (FFP) were performed at an RBC-to-FFP ratio of 2:1 to 1:1.

**Results:**

Over a 12-month investigation period, 123 severe multiple trauma patients needing intensive care therapy were admitted to our trauma center (mean age 48 years, mean ISS (injury severity score) 30). Fourteen (11%) patients died; 25 (mean age 51.5 years, mean ISS 53) of the 123 patients were treated using the “coagulation box,” and 17 patients required massive transfusions. Patients treated with the “coagulation box” required an average dose of 16.3 RBC and 12.9 FFP, whereas 17 of the 25 patients required an average dose of 3.6 platelet packs. According to the algorithm, 25 patients received fibrinogen (average dose of 8.25 g), 24 (96%) received PCC (3000 IU.), 14 (56%) received desmopressin (36.6 µg), 13 (52%) received tranexamic acid (2.88 g), and 11 (44%) received factor VIIa (3.7 mg). The clotting parameters markedly improved between SR admission and ICU admission. Of the 25 patients, 16 (64%) survived. The revised injury severity classification (RISC) predicted a survival rate of 41%, which corresponds to a standardized mortality ratio (SMR) of 0.62, which implies a higher survival rate than predicted.

**Conclusions:**

An Hb-driven algorithm, in combination with the “coagulation box” and the early use of clotting factors, could be a simple and effective tool for improving coagulopathy in multiple trauma patients.

## 1. Background

Massive hemorrhage is the leading cause of death in the first few hours following severe multiple traumas (1-3). Hemorrhage-related clotting disorders are common problems in this population of patients, particularly when the patients are hemodynamically unstable and require fluid resuscitation (2). For these reasons, early and aggressive treatment for clotting disorders is believed to reduce mortality. Point-of-care diagnostic devices (POCs), such as thromboelastography and thrombelastometry (e.g., TEG^®^ or ROTEM^®^), are not available in all trauma centers, although there is some evidence that they can improve outcomes (4). The results from commonly performed clotting studies are often delayed by at least 30 - 45 minutes. In time-sensitive emergency settings, this delay might be too long for appropriate therapeutic treatments. One parameter that is quickly available in the resuscitation room is the hemoglobin level. If a multiple trauma patient has already received fluid resuscitation, a certain relationship can be observed between the hemoglobin value and the development of clotting disorders (5-7).

## 2. Objectives

We have developed an Hb-driven algorithm to initiate the initial clotting therapy. The algorithm contains three different steps for aggressive clotting therapy (depending on the first Hb value measured in the shock trauma room, SR) and utilizes fibrinogen, prothrombin complex concentrate (PCC), factor VIIa, tranexamic acid and desmopressin. In this study, we evaluated the use of initial hemoglobin (Hb) levels as a guide line for the initial treatment of clotting disorders in multiple trauma patients guided by a specific algorithm, although this protocol is not recommended by the current European guidelines (8).

## 3. Patients and Methods

**Figure 1.**
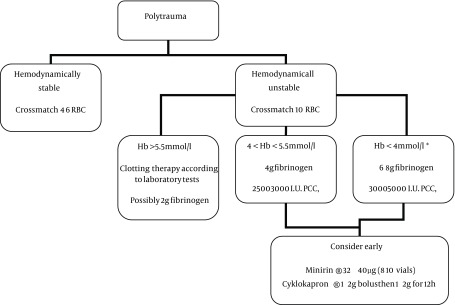
Standard Operating Procedure Diagram Abbreviations: FFP, fresh frozen plasma; RBC, red blood cells; PP, platelet pack; PCC, prothrombin complex concentrate, b If the bleeding continues after 30 minutes consider giving another 2 g fibrinogen and 3 mg Factor VIIa (Novo-Seven^®^). If after the second dose of F VIIa diffuse bleeding persists, consider 1 - 2 mg F VIIa continuously for 12 - 24 h.

From our experience with more than 120 multiple trauma patients (ISS > 16) per year and based on the clear increase (from approximately 4% to approximately 15%) in trauma patients requiring massive transfusions (defined as ≥ 10 units red blood cells (RBC) within 24 h), we have learned much about the relationship between the initial hemoglobin value and the extent of clotting disorders in patients who have already been given fluid resuscitation. Data from the literature and our own data show a good correlation between the initial hemoglobin value and clotting parameters, such as thromboplastin time (Quick value) (5-7, 9-11). For this reason and in the absence of point-of-care diagnostics (POCs) such as ROTEM^®^ in the SR, we have developed a hemoglobin-driven algorithm (described below) to guide the initial coagulation therapy. Despite the initial therapy with clotting factors, we transfused RBC and fresh frozen plasma (FFP) by estimating an RBC-to-FFP ratio of 2:1, as recommended by the literature (12). At our trauma center, coagulation therapy is conducted by the anesthetist on the trauma team. The following standard operating procedure (SOP) (Figure 1) was used for all trauma patients, and hemoglobin-driven coagulation therapy was administered to hemodynamically unstable patients.

### 3.1. Basic Instructions

-Stop the bleeding as soon as possible.

-Avoid or treat the “lethal triad” of hypothermia, acidosis and coagulopathy as early as possible.

-Administer RBCs and FFP at a 2:1 to 1:1 ratio.

-Manage initial therapy with coagulation factors that correspond to the initial hemoglobin value, clinical course, and possible source of bleeding.

### 3.2. Hb-Driven Clotting Therapy 

#### 3.2.1. Admission Hb >5.5 mmol/L

In these cases, the coagulability should be sufficient, for the most part. Manage coagulation therapy on the basis of the laboratory tests, and if in doubt, administer 2 g of fibrinogen. Always cross-match 10/10 RBC/FFP.

#### 3.2.2. mmol/L > Admission Hb < 5.5 mmol/L

In these cases, a clotting disorder might have already developed in the patients. Administer 2 - 4 g of fibrinogen and 2500 - 3000 IU of prothrombin complex concentrate (note the off-label use).

Always cross-match 20/20 RBC/FFP and 4 platelet packs. If there is any sign of hyperfibrinolysis, consider administering tranexamic acid as a 1 - 2 g bolus and 1 - 2 g continuously for 12 hours. If the patient has received HES or is on platelet aggregation inhibitors, consider administering desmopressin at 0.3 - 0.4 µg/kg BW. Closely monitor the plasma calcium.

#### 3.2.3. Admission Hb <4 mmol/L

In these cases, coagulability is likely to be severely compromised, and swift and aggressive therapy is of the utmost importance. Administer 4 - 6 g of fibrinogen, 3000 - 5000 IU of pro-thrombin complex concentrate (note the off-label use) and 1 mg activated factor VII (Novo-Seven^®^) (off-label use). Always cross-match 25/25 RBC/FFP and 6 platelet packs.

If there is any sign of hyperfibrinolysis, consider administering tranexamic acid as a 1 - 2 g bolus and 1-2 g continuously for 12 hours. If the patient has received HES or is on platelet-inhibiting drugs, consider administering desmopressin at 0.3 - 0.4 µg/kg BW. If the bleeding continues 30-60 minutes after treatment, consider administering another 2 g of fibrinogen and 3 mg factor VIIa. If diffuse bleeding persists after the second dose of factor VIIa, consider administering 1 - 2 mg NovoSeven^®^ continuously for 12-24 hours. Closely monitor the plasma calcium.

### 3.3. Coagulation Box

The drugs required for sufficient clotting therapy were stored in a special “coagulation box” in the hospital pharmacy, and this box was immediately brought to the SR or operation theater upon request. The hospital stored three of these boxes to ensure appropriate management in the event of simultaneous multiple trauma admissions. The “coagulation box” contains 5000 I.U. PCC, 10 g fibrinogen, 4 mg factor VIIa, 5 g tranexamic acid, 40 µg desmopressin and a documentation sheet. After the emergency room resuscitation, initial operative treatment proceeded according to the damage control concept, and intensive care unit (ICU) admission followed; all of the patients received continuous intravenous thromboembolism prophylaxis with standard heparin.

### 3.4. Data Collection

All the relevant data from multiple trauma patients receiving intensive care at our center have been reported anonymously into the Trauma Registry of DGU (Deutsche Gesellschaft für Unfallchirurgie/German Society of Trauma Surgery). Data collection and interpretation was approved by the Ethical Review Board of DGU, which waived the need of a written informed consent. After the introduction of the SOP as described above, we prospectively collected anonymous data from the patients who were treated according to the SOP and who required the “coagulation box.” The following data were collected:

- Laboratory tests, including hemoglobin, platelet count, Quick value (thromboplastin time), partial thromboplastin time (PTT), INR, D dimer, fibrinogen, base access and lactate.

- Scoring systems, including GCS (Glasgow Coma Scale) at the scene of the accident, ISS (Injury Severity Score), RISC (Revised Injury Severity Classification) and TASH score (Trauma Associated Severe Hemorrhage).

- The administration of RBCs, FFP, (apheresis) platelet packs, fibrinogen, prothrombin complex concentrate, activated factor VII, desmopressin, tranexamic acid and the amount of volume resuscitation.

- The development of thromboembolic events.

The main objective parameter was survival, which was based on the survival rate predicted by the Revised Injury Severity Classification (RISC) (13). The second objective parameter was coagulopathy improvement, as measured by the global standard tests. Data (lab results) were compared using the Students t-test. We applied a significance level of P < 0.05 to all statistical tests. Statistical analysis was performed using the standard statistical software (SPSS Chicago, IL, USA).

## 4. Results

From July 2011 to June 2012, 123 multiple trauma patients needing intensive care therapy were admitted to our trauma center. Their mean age was 48 years (Min 13 / Max 93), the mean ISS was 30, and 73% were male. Fourteen (11%) of the 123 patients died. Table 1 shows an overview of the 123 patients.

**Table 1.** Basic Information Regarding the 123 Multiple Trauma Patients

Information		
**Male, %**	78
**Age, y**	48 (min 13/ max 93)
**ISS^a^**	30
**RISC^a^ (predicted mortality)**	21
**Mortality**	11
**Traffic accident**	58
**Fall**	35
**Suicide**	6
**Violent crime**	2
**Penetrating trauma**	2
**Shock on admission**	25
**Head injury (AIS^a^ ≥ 3**)	59
**Intubated on admission, %**	58
**On-scene CPR^a^**	2
**RBC^a^ transfusion required**	34
**Massive transfusion (≥ 10 RBC^a^**)	14

^a^Abbreviations: AIS, abbreviated injury scale CPR, cardio-pulmonary resuscitation; ISS, injury severity score; RBC, red blood cell; RISC, revised injury severity classification

Twenty-five (27%) of the 123 patients were treated with the “coagulation box”. Of the 25 patients, 72% were male, and the average age was 51.5 years. The mean GCS at the scene of the accident was 6.4, and the mean ISS was 53. All 25 patients required RBC transfusions, and 17 (68%) required massive transfusions (≥ 10 RBC). Table 2 shows basic information about the 25 patients who required early coagulation therapy with the “coagulation box”.

**Table 2.** Basic Information Regarding the Patients Requiring the “Coagulation Box”

Information		
**Male, %**	72
**Age, Mean, y**	51.5
**ISS** ^a^	53
**RISC ^a^ (predicted mortality)**	59,2
**Mortality, %**	36
**Shock on admission**	96
**GCS ^a^ on scene**	6.4
**Intubated on admission**	96
**On-scene CPR** ^a^	8
**Massive Transfusion (≥10 RBC ^a^)**	68

^a^Abbreviations: CPR, cardio-pulmonary resuscitation; GCS, glasgow coma scale; ISS, injury severity score; RBC, red blood cell; RISC, revised injury severity classification

The 25 patients treated with the “coagulation box” received average doses of 16.3 RBC (4 minimum, 40 maximum) and 12.9 FFP (4 minimum, 30 maximum), and 17 of the 25 patients required platelet transfusions, with an average dose of 3.6 platelet packs (2 minimum, 8 maximum). As per the SOP, all 25 patients received fibrinogen (8.25 g average dose), and 24 patients received prothrombin complex concentrate (3000 I.U. average dose). Fourteen patients received desmopressin (36.57 µg average dose); 13 patients received tranexamic acid (2.88 g average dose); and 11 patients received activated factor VII (3.7 mg average dose). Balanced crystalloid fluid resuscitation (emergency room and operating room) was provided to all patients in an average dose of 8895 mL, and colloids were strictly avoided. Table 3 lists the doses of the administered drugs and blood products, and the volume of fluid resuscitation.

**Table 3.** Average Doses of the Administered Drugs and Blood Products and Volume of Fluid Resuscitation

	Patients, No. (%)	Average Dose	Min / Max	SD
**Crystalloids, mL**	25 (100)	8895	3000 / 17000	4441
**RBC^a^, packs**	25 (100)	16.3	4 / 40	8.15
**FFP^a^, packs**	24 (96)	12.9	4 / 30	7.8
**Platelet, packs**	17 (68)	3.6	2 / 8	1.64
**Fibrinogen, g**	25 (100)	8.25	4 / 16	3.47
**PCC ^a^, IU**	24 (96)	3000	1000 / 5000	1330
**Desmopressin, µg**	14 (56)	36.57	28 / 72	10.6
**Tranexamic acid, g**	13 (52)	2.88	1 / 5	1.64
**NovoSeven^®^, mg**	11 (44)	3.7	1 / 10	2.56

^a^Abbreviations: FFP, fresh frozen plasma; PCC, prothrombin complex concentrate; RBC, red blood cell

Of the 25 patients, 16 (64%) survived. The RISC predicted a survival rate of 41%, which corresponded to an SMR (standardized mortality ratio) of 0.64 and implied a higher survival rate than expected. Five patients died from uncontrolled bleeding within the first 24 hours despite all efforts; two patients died from multi-organ failure during the critical care stage and two from traumatic brain injury. The TASH score predicted a massive transfusion (≥ 10 RBC) rate of 40.3%, which was much lower than the observed rate of 68%. Fifteen of the patients who were treated with the “coagulation box” had an initial Hb < 5.5 mmol/L, and 6 had a Hb < 4 mmol/L. Ten patients were treated with the “coagulation box” because of their clinical courses and laboratory results (measured in the emergency room), despite an initial Hb > 5.5 mmol/L. A thromboembolic event occurred in two of the 25 patients. One survived after developing a grade II pulmonary embolism caused by deep leg vein thrombosis, and one patient died from multi-organ failure after developing multiple small vessel thrombosis. Table 4 lists the results of the initial laboratory tests performed at admission to the resuscitation room and the results obtained at the time of ICU admission.

**Table 4.** Laboratory Results

Lab Test	Resuscitation Room, Mean ± SD	ICU Admission, Mean ± SD	P Value
**Hemoglobin, mmol/L**	5.4 ± 2.2	6.1 ± 1.44	n.s^a^
**Platelet count, Gpt/L**	155 ± 94	98 ± 50	< 0.05
**Quick value, %**	58 ± 29.2	97 ± 24.4	< 0.001
**PTT ^a^, s**	54.2 ± 38.4	41 ± 14.3	n.s^a^
**INR** ^a^	1.66 ± 0.9	1.1 ± 0.25	< 0.005
**D dimer, mg/L**	24.2 ± 13.5	13.4 ± 14.9	< 0.05
**Fibrinogen, g/L**	1.58 ± 0.82	2.17 ± 0.69	< 0.01
**BE ^a^, mmol/L**	-7.5 ± 2.31	-2.9 ± 4.6	< 0.001
**Lac ^a^, mmol/L**	5.5 ± 4.16	5.8 ± 3.4	n.s^a^

^a^Abbreviations: BE, base excess; INR, international normalized ratio; Lac, lactate; n.s , not significant; PTT, partial thromboplastin time

## 5. Discussion

Coagulopathy is a common finding in patients with multiple traumas. Up to 34% of multiple trauma patients already have some form of coagulopathy at the time of admission to the shock trauma room (11). In an aging population, more patients are likely to be taking anticoagulants, such as warfarin, aspirin, clopidogrel, the new oral direct factor Xa and thrombin inhibiters; therefore, the percentage of multiple trauma patients with coagulopathy in the shock trauma room is also likely to increase. Although coagulability is only slightly impaired in many cases, there are cases in which coagulability is severely compromised and thus, quick and aggressive therapy is of utmost importance. In these cases, for the purposes of establishing adequate coagulation therapy, it is desirable to have clotting studies that reveal the extent of clotting disorders within a few minutes. Even point of care (POC) devices, such as TEG^®^ or ROTEM^®^, require some time (at least 10 - 15 minutes), and standard clotting studies are even more time consuming. POC devices, also require additional personnel; even German level one trauma centers are unable to provide staff around the clock. In the literature, a number of studies on bleeding in multiple trauma patients have revealed a relationship between the initial clotting disorders and the initial hemoglobin values (7, 9-11). By comparing four groups of trauma patients with different levels of fluid resuscitation, Hussmann et al. (10) found that the course of hemoglobin and the Quick value were related. Therefore, it seems reasonable to use the initial hemoglobin value (as a quickly available parameter) to determine the initial clotting therapy for a bleeding, hemodynamically unstable patient. The results obtained from our trauma patients showed a clear correlation between the initial hemoglobin value and the Quick value (5, 6). Although the “European guideline for management of bleeding following major trauma” (8) does not recommend using Hct, which corresponds with hemoglobin, as a marker for bleeding, we developed the described hemoglobin-driven algorithm for the purposes of initiating early and aggressive clotting therapy. A recent study with a large trauma cohort showed that hemoglobin and Hct levels at the time of admission were predictors of mortality within the first seven days (14). The described SOP using Hb to initiate early clotting therapy and the “coagulation box” were deemed to be practicable and easy to use by the entire trauma team. Our results revealed a lower mortality rate than predicted by the RISC, which we believe supports the efficiency of the described SOP and the “coagulation box.” However, in light of the small number of patients, this interpretation should be viewed with caution. The massive transfusion requirement was 68%, which was much higher than that predicted by the TASH score. One reason for this higher transfusion requirement is that four patients were admitted to the shock trauma room within 45 minutes of their accidents and only minimal fluid resuscitation was performed. Although the patients were unstable at the time of admission, two of the important parameters for calculating the score (hemoglobin and base excess) were not as unstable as it may have been expected. The scores were developed and validated using data from the German Trauma Registry (15, 16), and the scores allowed for the early and reliable estimation of the probability of mass transfusion with a good result in a large cohort. In the subgroup of trauma patients presented here, the score clearly underestimated the probability of mass transfusion. Some of our algorithm recommendations require a more detailed examination. The first clotting factor used in our algorithm was fibrinogen. This approach is reasonable because fibrinogen is the first factor that reaches critically low values in a bleeding patient (8, 17, 18). Additionally, fibrinogen administration has been associated with a reduction of blood product transfusion (19) and with improved survival in patients with combat-related traumas (20). In our second step, we used prothrombin complex concentrate (PCC), which is much more questionable than the use of fibrinogen. The current “European guideline for the management of bleeding following major trauma” (8) does not support the use of PCC in bleeding trauma patients unless they have taken vitamin K antagonists. Although PCC is not recommended by the current guidelines, evidence suggests that the use of PCC in bleeding patients is ingenious (18, 21-23). Several animal studies have demonstrated the effectiveness of PCC in correcting dilutional coagulopathy (22, 24), and reports in humans have described successful PCC use in bleeding patients (18, 21). The combination of fibrinogen and PCC could be promising and has already been used successfully. Schöchl et al. (25) compared thromboelastometry-guided hemostatic therapy with fibrinogen concentrate and PCC in trauma patients at the Salzburg Trauma Center (Austria); patients from the Trauma Register DGU received ≥ 2 units of FFP but no fibrinogen concentrate or PCC. A significant reduction in the amounts of RBCs and platelets transfused was observed, with a slight decrease in mortality rate. Unlike the European guidelines, the “Cross-Sectional Guidelines for Therapy with Blood Components and Plasma Derivatives,” which was published by the Board of the German Medical Association on the Recommendation of the Scientific Advisory Board (26, 27), and the current “Poly-trauma” guideline from the German Trauma Society (28) have recommended the use of PCC in bleeding patients. The German Medical Association stated that in cases of severe disseminated intravascular coagulation and loss or dilution coagulopathy, a prothrombin complex deficiency could be pronounced to such a degree that, in addition to FFP infusion, substitution with PCC will be required (26). The third approach towards unstable, bleeding trauma patients was the early use of NovoSeven^®^, although at a relatively different dose than what is usually described in the trauma literature. The dose recommendation for NovoSeven^®^ in the trauma population varies from 40 µg/kg BW (29) to 200 µg/kg BW (30), which corresponds to 3-15 mg for a 75 kg patient. If such a high dose (90 µg/kg BW and more) is used, the Quick value will greatly exceed the upper limit, creating the theoretical danger of thromboembolic events. Levi et al. (31) found a correlation between the factor VIIa dose and thromboembolic events. In our approach, we attempted to maintain the Quick value within the upper limit when using the doses that are recommended in our SOP. The second reason for choosing a much lower factor VIIa dose than recommended in the literature, was that the level of factor VII with the administered PCC was already increased and some theoretical points led us to believe a lower factor VIIa dose than recommended in the literature was sufficient in these cases; even the effect of supra normal doses of factor VIIa is the bypass activation of factor X (32). The combination of PCC and a full dose of factor VIIa could dramatically increase thromboembolic events. As shown in Figure 1, our SOP recommends the early consideration/use of tranexamic acid and desmopressin. The rationale for the first recommendation is quite clear. Many trauma patients initially develop hyperfibrinolysis during the first hours of resuscitation, particularly patients with serious traumatic brain injuries, middle face fractures or serious thoracic injuries. Hyperfibrinolysis is often considered to be a direct consequence of the combination of tissue injury and shock (33). Therefore, tranexamic acid could be a useful drug for bleeding multiple trauma patients in the early stages of treatment. Regarding the results of the CRASH II trial, there is evidence that tranexamic acid reduces mortality in bleeding trauma patients while it has been shown that it must be administered within the first three hours of admission (34, 35). The evidence for using desmopressin in bleeding multiple trauma patients is much weaker; however, there have been some clues suggesting that desmopressin might be useful (36, 37), despite the current European guidelines that do not recommend desmopressin for routine use (8). Some drugs taken by the elderly trauma population, such as aspirin or clopidogrel, and some fluids used for resuscitation, such as hydroxyethyl starch, interact with platelet function. In these cases, desmopressin appears to be a useful drug for restoring platelet function (38). Despite the evidence for the use of the clotting factors and the drugs described above, our results showed clear improvement in the measured global clotting studies, with almost normal values at the time of ICU admission. Even D dimer, which is considered an indirect sign of hyperfibrinolysis, was clearly lower. Unfortunately, we were unable to measure hyperfibrinolysis because of the lack of a ROTEM^®^ or TEG^®^. Hence, we used D dimer as a surrogate parameter. Regardless of the described SOP, our results revealed that the “coagulation box” was used wisely. A massive transfusion requirement of 68% and the average requirements of 16.3 RBCs, 12.9 FFP and 3.6 platelet packs are good indicators of the correct use of the “coagulation box.” The box, in combination with the SOP, was a simple and effective tool that provided the trauma team with the necessary drugs to treat bleeding trauma patients. Irrespective of the “coagulation box,” the basis for the successful treatment of a bleeding trauma patient is volume resuscitation, blood and plasma transfusion and an early surgical approach to terminate the bleeding.

### 5.1. Limitations

Our investigation had some limitations. The main limitation was the small number of patients, however, the number of patients evaluated will increase over time. A second limitation was the uncontrolled nature of the investigation. We did not have a control group to verify the efficiency of our approach. Our conclusion that the described SOP, in combination with the “coagulation box”, was a simple and effective tool was based on the clear reduction in mortality compared with the mortality predicted by the RISC. The investigation did not assess any outcome parameters beyond the mortality and laboratory results. Our SOP leads to some recommendations that are not supported by all guidelines, but as discussed earlier, the recommendations are supported by clinical data and by physiological and pathophysiological considerations; therefore, they should be clinically rational. The use of the first hemoglobin value measured in the trauma room as a surrogate parameter for any form of coagulopathy is not supported by studies that have focused on this issue. However, as mentioned above, if the hemoglobin values and the corresponding thromboplastin time/Quick/INR values in the trauma literature are considered, a stable correlation between these two parameters can be established. Implementing early and aggressive clotting therapy in an unstable, bleeding trauma patient is an important goal and can reduce mortality rates beyond than RISC predictions. Using a hemoglobin-driven SOP and three different levels of clotting therapy (determined by the initial Hb value and the clinical course of the patient) is a simple means of achieving this goal (early and aggressive clotting therapy). A “coagulation box” that contains all of the clotting agents required for a bleeding trauma patient can quickly provide the shock trauma team with all of the drugs required to initiate the appropriate clotting therapy. Further investigations are needed to clarify the role of the proposed algorithm in bleeding trauma patients. Despite the results of laboratory studies, surgical bleeding control should be obtained as early as possible.

The following points are the key findings of our work:

- There is a stable correlation in the literature between the admission hemoglobin levels and the results of the standard clotting studies in bleeding trauma patients.

- An Hb-driven algorithm to initiate clotting therapy is simple and effective.

- The “coagulation box” provides the trauma team with all of the drugs required for the appropriate management of bleeding patients.

- Early and aggressive clotting therapy, according to the described algorithm, could reduce mortality and improve upon standard clotting studies.

- Trauma-related coagulopathy should be treated “aggressive and early.”
